# Did the COVID-19 Pandemic Spark a Public Interest in Pet Adoption?

**DOI:** 10.3389/fvets.2021.647308

**Published:** 2021-05-07

**Authors:** Jeffery Ho, Sabir Hussain, Olivier Sparagano

**Affiliations:** ^1^Department of Infectious Diseases and Public Health, Jockey Club College of Veterinary Medicine and Life Sciences, City University of Hong Kong, Hong Kong, China; ^2^Department of Epidemiology and Public Health, University of Veterinary and Animal Sciences, Lahore, Pakistan

**Keywords:** COVID-19, Google trend, pet adoption, companion animal, animal welfare, epidemiology, public health, infodemiology

## Abstract

This study aimed to determine if there has been an increase of global interest on pet adoption immediately after the WHO declaration of the pandemic and if the effect has been sustainable in 8 months on. We conducted a Google Trends search using keywords related to pet adoption. Relative search volume (RSV) was scored between 0 and 100 for the lowest and the highest, respectively. Top countries contributing to the dataset included Australia, the United States, Canada, New Zealand, the United Kingdom, Singapore, the Philippines, and Malaysia. From 2015 through 2020, the worldwide RSV for the categories of pet, dog and cat adoption peaked between April and May 2020, the early epidemic phase of the pandemic. These were significantly higher than the 5-year worldwide average RSV for all three categories (*P* = 0.001). Comparing to the same period in 2019, the RSV ratio (2020/2019) for both dog and cat adoption increased by up to 250%. Nonetheless, the RSV for dog adoption has been decreasing since July 2020 and returned to the 5-year average by December 2020. In contrast, the interest in cat adoption remained sustainably high, possibly reflecting the feline acclimation to indoor living. In conclusion, the global interest in pet adoptions surged in the early phase of the pandemic but not sustainable. With the launch of COVID-19 vaccines, there is a concern for separation anxiety and possible abandonment of these newly adopted pets when the owners would leave their homes for work in the future.

## Introduction

Cats and dogs are amongst the common domesticated animals for human companionship, and their bonds with humans generally provide mutual psychosocial health benefits (1). Following the global spread of the COVID-19 pneumonia, social distancing measures such as working from the home policy has been reinforced worldwide (2, 3). Along with an increased unemployment rate, the average hours per day spent at home for the general population has considerably increased after the pandemic as compared to the corresponding seasons in 2019 (4). Sporadic news reports have indicated an increase of dog and cat adoption from animal shelters (5–8). The United Kingdom has even witnessed an emptied shelter because of an ever-high adoption rate (5). That being said, abandonment of pet dogs and animal cruelty have also been reported amidst the pandemic (9), probably due to rumors of animals as potential reservoirs of COVID-19 (10, 11). Stray dogs were starved and some of them were subsequently euthanized (12). Animal cruelty is widespread and has been reported in developed and developing nations (13). In Hong Kong, there were 20 people arrested for animal cruelty during the first half of the year 2020, compared to 36 arrests over the whole year in 2019 (14).

Google Trends™ is a freely accessible database of worldwide internet searches and has been widely used for identifying population interest on health and social issues (15–17). Comparing to conventional epidemiologic surveys, Google Trends provide real-time data without concern for recall bias and reporting bias (16). In fact, the use of the internet data for disease surveillance and health behavior could be traced back to early 2000s when the term of infodemiology was introduced to define web-based epidemiology studies (18). In this study, we aimed to investigate the global interest in pet adoption during the COVID-19 pandemic.

## Methods

The Google Trend (GT) is a well-established freely available source of big data for temporal analysis of information-seeking behavior of the general public (19–22). The relative search volumes (RSV) between 1st December 2015 and 1st December 2020 were collected for the search terms in the categories of pet adoption (“adopt a pet,” “pet adoption near me,” “adoption center”), dog adoption (“dog adoption,” “puppy adoption,” “dogs for adoption near me”), and cat adoption (“cat for adoption,” “kittens for adoption,” “cats for adoption near me”). The RSV ranges from 0 to 100 and is normalized to the total search volume according to regions, rendering it insensitive to population size and regional accessibility to the internet. This allows direct comparison of RSV between geographical locations. As such, a value of 100 denotes the peak popularity between the above-stated search period, whereas 0 represents RSV below a detectable level over the enquired time period. An asterisk wildcard was added to represent truncated words as appropriate. Putative search terms were chosen using the function of related queries in GT. Candidate search terms were those with RSV greater or equal to 30% of the total. The consensus was reached by discussion among authors. Country-specific data of the top contributing countries to the RSV were also obtained using customizable filters. All data from GT was then exported to a comma separated value (CSV) file which was then imported into Statistical Package for Social Sciences (SPSS version 25.0) for statistical analysis. Statistical significance was set at 0.05. All analysis was two-tailed.

Kolmogorov-Smirnov test for normality was used to determine if the RSV dataset was normally distributed across the search period (23). For normally distributed datasets, the differences of continuous variables between two groups were compared using independent Student's *t*-test. Otherwise, the dataset would either be logarithmically normalized or analyzed by non-parametric Mann–Whitney *U* test. The mean RSV of each month between 1^st^ December 2015 and 1^st^ December 2020 was computed. The monthly mean RSV between December 2015 and November 2019 was compared to that between December 2019 and December 2020 compared with RSV in other periods of interest (RSV ratio).

## Results

### The Overall Popularity of Pet Adoption in Different Geographical Regions

Between 1^st^ December 2015 and 1^st^ December 2020, the public interest on pet adoption was expressed as a fraction of total searches in a specific geographical region. The RSV ranked the highest in the United States (100) followed by Australia (52), Canada (46), Singapore (28), and the United Arab Emirates (27). Countries ranked from the fifth to the tenth had an overall RSV <12. These included New Zealand, Malaysia, the United Kingdom, India and Ireland. For dog adoption, regions in descending order of public interest were the United States (100), Australia (99), Singapore (93), Canada, New Zealand (44), United Kingdom (34), United Arab Emirates (33), Ireland (32), Malaysia (20) and Hong Kong (20). For cat adoption, Singapore had the highest popularity (100) followed by the United States (68), Canada (65), Australia (63), United Kingdom (44), United Arab Emirates (43), New Zealand (32), Hong Kong (27), Ireland (23), and Malaysia (19).

### Immediate and Long-Term Effect of COVID-19 on Public Interest

**Figure 1:**
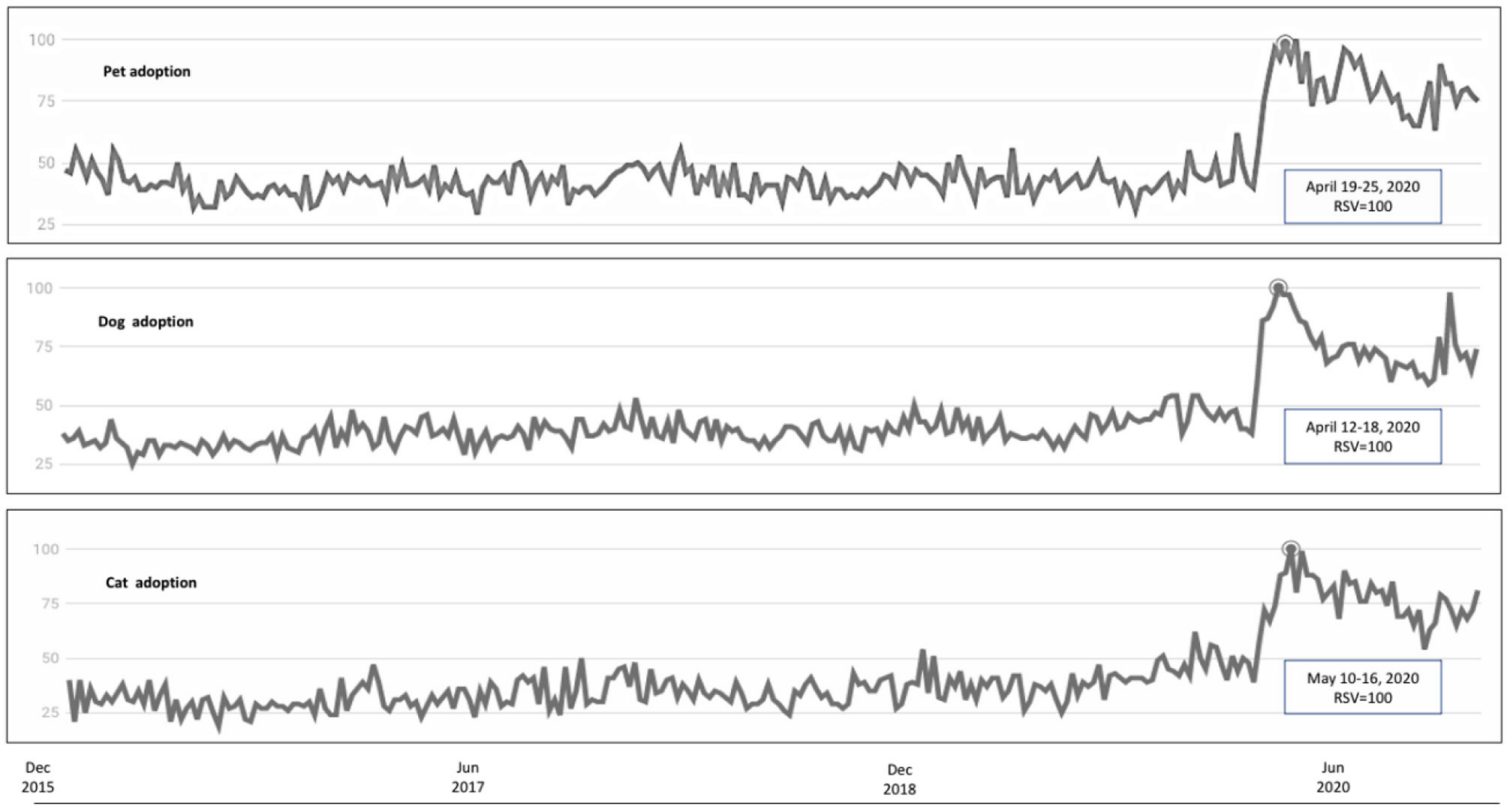
Relative search volume of pet adoption related key words between December 2015 and December 2020. The panels from top to bottom indicated the searches for pet adoption, dog adoption, and cat adoption, respectively. Peaks as indicated by rectangles were observed following the WHO declaration of the COVID-19 pandemic.

From 2015 to 2020, the worldwide relative search volume (RSV) for dog adoption and cat adoption peaked at April 2020, the early epidemic phase of the COVID-19 pandemic (Figure 1). Likewise, the interest in cat adoption peaked at 10^th^-16^th^ May 2020. These were significantly higher than the 5-year worldwide RSV average for pet (40), dog (58) and cat (21) (Mann–Whitney *U* test, *P* = 0.001). Interestingly, the RSV for cat adoption remained sustainably high through December 2020 whereas that for dog adoption has been gradually decreasing to 50, a level comparable with the 5-year average. Comparing the worldwide RSV between 2020 and 2019 on the same month, the RSV ratio (2020/2019) for both dog (1.42, range= 1.18–1.91) and cat adoption (1.90, 1.55–2.54) has been increased by up to 250%. In descending order, the contributing search volume in Australia was the highest (RSV = 100), followed by the United States, Canada, New Zealand, and the United Kingdom (RSV range = 42–90). In Asia, search for pet adoption was the highest in Singapore (RSV = 38), followed by the Philippines, and Malaysia (RSV range = 20–36).

## Discussion

It has been questioned that cats and dogs may carry COVID-19 virus which in turn act as a reservoir for infection for pet owners (24, 25). Nonetheless, no evidence has clearly indicated that these pet animals have been implicated in zoonosis events of COVID-19 and therefore it should be safe to adopt cats and dogs as well as to keep these pet animals in their home (2, 3). Besides, still One health approach along with follow up of good hygiene, sanitation and cleanliness need to be kept as like preventive measures which would also aid in maintaining disease free environments for these pet animals as public health intervention strategies. Surveillance and monitoring of animals need to be conducted by health agencies to rule out any possibility of SARS coV-2 as well as strengthen the beliefs of pet-owners that it is safe to own or adopt these pet animals. Additionally, lock-down confinement during COVID-19 pandemic pets provided good emotional support to their owners as usual as members of family. Prioritizing pets' welfare can impact the health and well-being of their owners (26–28).

The COVID-19 pandemic has brought about a considerable impact on animals for food production and for companions (27–34). Our study revealed that the web interest on adoption of cats and dogs increased during the early phase of the COVID-19 pandemic (35) and appeared to be sustainable for that of cats but not dogs. The affection between human and companion animals is well-recognized as an effective way to minimize stress during an uncertain period and help alleviating depression and anxiety upon social isolation (7, 26, 36). In face of disasters, the majority of pet owners are not willing to surrender their companion animals (37). Although there are reports of increasing pet abandonment during the pandemic, it appeared that the increased rates of adoption have canceled out the number of surrendered pets (7, 10). Other than adoption, studies have also reported an increase in the number of foster families for the temporary stay of animals pending for adoption and a shorter length of stay for animals in shelters (6, 7).

While the pandemic provides a unique time for building bonds between the owners and the newly-adopted animals, there are concerns that these pets may experience separation anxiety or be returned to the shelter when the owners are no longer working from home (5, 8, 38). Following the close bonding established with their owners during the pandemic, their dogs and cats could experience separation anxiety due to sudden disappearance of their human companions. A period of hyperattachment between owners and pets, such as that seen in the current pandemic, is an established risk factor for separation anxiety in dogs (39). Separation anxiety could result in excessive barking, aggression and destructive behavior such as damaging the indoor furniture when the owners are away for work. These behavioral problems of the pet animals may be overwhelming to inexperienced pet owners who adopt their pets for the first time during the pandemic. In fact, the concern for pet abandonment after the pandemic has been acknowledged by animal shelter organizations (8). Following the announcement of estimated dates of introduction of the COVID-19 vaccines, the public interest in dog adoption has considerably reduced in October and November to a level comparable to the 5-year average.

Although pet adoption rates have been increased during the pandemic, there were concerns for abandonment of pets and shortage of veterinary care that may have impacted the welfare of these animals. Studies have indicated that pets were abandoned because of concern for carriage of COVID-19 (10, 40) although the likelihood for companion animals in spreading COVID-19 remains low (24, 25, 41). Some of the pet owners may become reluctant to visit veterinary clinics and thus precluding the pets from elective veterinary procedures that could have an impact on the animal physical health and well-being. Euthanasia of pets may have increased, albeit there is no data available on this aspect. It has been reported that veterinary care products and services were under shortage in food animal production industry due to the pandemic (34). It could be speculated that the availability of veterinary drugs may have become limited in some of the countries.

This study is limited by the fact that relative search volume may not reflect the general interest of all population in the world and that web interest does not necessarily lead to effective actions.

## Conclusion and Recommendations

We observed a global interest in pet adoption, in particular for dogs, following the COVID-19 pandemic. Considering the launch of COVID-19 vaccines, the animals may experience separation anxiety when their owners would return to work. Special training or awareness campaigns for pet owners may be required during the transition period.

## Data Availability Statement

The original contributions generated for the study are included in the article/supplementary material, further inquiries can be directed to the corresponding author/s.

## Author Contributions

JH conceived this study, conducted literature search, statistical analysis, and wrote the manuscript. SH and OS provided intellectual input, assisted data curation, and conducted literature review. All authors agreed with the final version of the manuscript for publication.

## Conflict of Interest

The authors declare that the research was conducted in the absence of any commercial or financial relationships that could be construed as a potential conflict of interest.

## References

[1] Wong PWC Yu RWM Ngai JTK. Companion animal ownership and human well-being in a metropolis—the case of Hong Kong. Int J Environ Res Public Health. (2019) 16:1729. 10.3390/ijerph16101729 31100852 PMC6571622

[2] Dhama K Patel SK Sharun K Pathak M Tiwari R Yatoo MI. SARS-CoV-2 jumping the species barrier: zoonotic lessons from SARS, MERS and recent advances to combat this pandemic virus. Travel Med Infect Dis. (2020) 37:101830. 10.1016/j.tmaid.2020.101830 32755673 PMC7396141

[3] Dhama K Khan S Tiwari R Sircar S Bhat S Malik YS. Coronavirus disease 2019-COVID-19. Clin Microbiol Rev. (2020) 33:e00028–e00020. 10.1128/CMR.00028-20 32580969 PMC7405836

[4] Hong Kong Census and Statistics Department. Unemployment and Underemployment Statistics for February to April (2020). Available online at: http://www.censtatd.gov.hk/hkstat/sub/sp200.jsp?productCode=B1050001 (accessed February 10, 2021).

[5] Barr S. Coronavirus Pandemic Sees Huge Increase in Cat and Dog Adoptions. (2020). Available online at: https://www.independent.co.uk/life-style/coronavirus-dog-cat-pet-adoption-battersea-rehome-covid-19-a9426741.html (accessed December 28, 2020).

[6] Grey EE. Thanks to Sheltering in Place, Animal Shelters are Empty. (2020). Available online at: https://www.wired.com/story/coronavirus-pet-adoption-boom (accessed December 28, 2020).

[7] Morgan L Protopopova A Birkler RID Itin-Schwar B Sutton GA Gamliel A. Human-dog relationships during the COVID-19 pandemic: booming dog adoption during social isolation. Humanit Soc Sci Commun. (2020) 7:155. 10.1057/s41599-020-00649-x

[8] Teenan T. Animal Shelters Have Welcomed the Coronavirus-Related Boom in Pet Adoption and Fostering. But Some are Also Planning for Large Numbers of Pets Being Returned and Financial Peril. (2020). Available online at https://www.thedailybeast.com/coronavirus-sparks-a-pet-adoption-and-fostering-boom-but-animal-shelters-worry-it-may-go-bust (accessed December 28, 2020).

[9] Vincent A Mamzer H Ng Z Farkas KJ. People and their pets in the times of the COVID-19 pandemic. Society Register. (2020) 4:111–28. 10.14746/sr.2020.4.3.0632612749

[10] Parry NMA. COVID-19 and pets: when pandemic meets panic. Forensic Sci Int. (2020) 2:100090. 10.1016/j.fsir.2020.100090 PMC7151387 38620282

[11] Huang Q Zhan X Zeng XT. COVID-19 pandemic: stop panic abandonment of household pets. J Travel Med. (2020) 27:taaa046. 10.1093/jtm/taaa046 32268360 PMC7184468

[12] Kim CNN. Cats and Dogs Abandoned at the Start of the Coronavirus Outbreak are Now Starving or Being Killed. (2020). Available online at: https://www.cnn.com/2020/03/15/asia/coronavirus-animals-pets-trnd/index.html (accessed December 28, 2020).

[13] Reese LA Vertalka JJ Richard C. Animal cruelty and neighborhood conditions. Animals. (2020) 10:2095. 10.3390/ani10112095 PMC7696964 33187259

[14] Sun F. More Animals Dumped, Abused Amid Covid-19 Pandemic in Hong Kong as Owners Leave or Say They Can't Afford to Keep Pets. (2020). Available online at: https://www.scmp.com/news/hong-kong/society/article/3108783/more-animals-dumped-abused-amid-covid-19-pandemic-hong-kong (accessed December 28, 2020).

[15] Nuti SV Wayda B Ranasinghe I Wang S Dreyer RP Chen SI. The use of Google Trends in health care research: a systematic review. PLoS ONE. (2014) 9:e109583. 10.1371/journal.pone.0109583 25337815 PMC4215636

[16] Bhambhvani HP Tijerina JD Parham MJ Grenberg DR Eisenberg ML. Public interest in elective urological procedures in the COVID-19 pandemic: a Google Trends analysis. Urology Practice. (2020) 7:496–501. 10.1097/UPJ.0000000000000179 37287180

[17] Heerfordt C Heerfordt IM. Has there been an increased interest in smoking cessation during the first months of the COVID-19 pandemic? A Google Trends study. Public Health. (2020) 183:6–7. 10.1016/j.puhe.2020.04.012 32388011 PMC7167577

[18] Eysenbach G. Infodemiology and infoveillane: framework for an emerging set of public health informatics methods to analyze search, communication and publication behavior on the internet. J Med Internet Res. (2009) 11:e11. 10.2196/jmir.1157 19329408 PMC2762766

[19] Cervellin G Comelli I Lippi G. Is Google Trends a reliable tool for digital epidemiology? Insights from different clinical settings. J Epidemiol Global Health, (2017) 185–9. 10.1016/j.jegh.2017.06.001 28756828 PMC7320449

[20] Mavragani A Ochoa G. Google trends in infodemiology and infoveillance: methodology framework. JMIR Public Health Surveill. (2019) 5:e13439. 10.2196/13439 31144671 PMC6660120

[21] Carneiro HA Mylonakis E. Google trends: a web-based tool for real-time surveillance of disease outbreaks. Clin Infect Dis. (2009) 49:1557–64. 10.1086/630200 19845471

[22] Mavragani A Ochoa G Tsagarakis KP. Assessing the methods, tools and statistical approaches in Google Trends Research: systematic review. J Med Int Res. (2018) 20:e270. 10.2196/jmir.9366 30401664 PMC6246971

[23] Justel A Pena D Zamar R. A multivariate Kolmogorov-Smirnov test of goodness of fit. Stat Probabil Lett. (1997) 35:251–9.

[24] Csiszar A Jakab F Valencak TG Lanszki Z Toth GE Kemenesi G. Companion animals likely do no spread COVID-19 but may get infected themselves. Geroscience. (2020) 45:1229–36. 10.1007/s11357-020-00248-3 32766998 PMC7410515

[25] Mallapaty S. Coronavirus can infect cats—dogs, not so much. Nature. (2020). 10.1038/d41586-020-00983-8. [Epub ahead of print]. 32238897

[26] Bowen J Garcia E Darder P Arguelles J Fatjo J. The effects of the Spanish COVID-19 lockdown on people, their pets, and the human-animal bond. J Vet Behav. (2020) 40:75–91. 10.1016/j.jveb.2020.05.013 32837452 PMC7292953

[27] Latif AA Mukaratirwa S. Zoonotic origins and animal hosts of coronaviruses causing human disease pandemics: a review. Onderstepoort J Vet Res. (2020) 87:e1–9. 10.4102/ojvr.v87i1.1895 33354975 PMC7756848

[28] Tiwari R Dhama K Sharun K Iqbal Yatoo M Malik YS Singh R. COVID-19: animals, veterinary and zoonotic linkes. Vet Q. (2020) 40:169–82. 10.1080/01652176.2020.1766725 32393111 PMC7755411

[29] Applebaum JW Adams BL Eliasson MN Zsembik BA McDonald SE. How pets factor into healthcare decisions for COVID-19: a one-health perspective. One Health. (2020) 11:100176. 10.016/j.onehlt.2020.100176 33062838 PMC7543786

[30] Bonilla-Aldana DK Dhama K Rodriguez-Morales AJ. Revisiting the One Health approach in the context of COVID-19: a look into the ecology of this emerging disease. Adv Anim Vet Sci. (2020) 8:234–7. 10.17582/journal.aavs/2020/8.3.234.237

[31] El Zowalaty ME Jarhult JD. A previously unknown SARS-related coronavirus (SARS-CoV-2) of pandemic potential infecting humans—call for a one health approach. One Health. (2020) 24:100124. 10.1016/j.onehlt.2020.100124 32195311 PMC7075990

[32] Hafez HM Attia YA. Challenges to the poultry industry: current perspectives and strategic future after the COVID-19 outbreak. Front Vet Sci. (2020) 7:516. 10.3389/fvets.2020.00516 33005639 PMC7479178

[33] Haider N Rothman-Ostrow P Osman AY Arruda LB Macfarlane-Berry L Elton L. COVID-19 zoonosis or emerging infectious disease? Front Public Health. (2020) 8:596944. 10.3389/fpubh.2020.596944 33324602 PMC7725765

[34] Hussain S Hussain A Ho J Sparagano OAE Zia UU. Economic and social impacts of COVID-19 on animal welfare and dairy husbandry in Central Punjab, Pakistan. Front Vet Sci. (2020) 7:589971. 10.3389/fvets.2020.589971 33195626 PMC7644897

[35] Holland KE Owczarczak-Garstecka SC Anderson KL Casey RA Christley RM Harris L. More attention than usual: a thematic analysis of dog ownership experiences in the UK during the first COVID-19 lockdown. Animals. (2021) 11:e240. 10.3390/ani11010240 33477947 PMC7833365

[36] Hoy-Gerlach J Rauktis M Newhill C. Non-human animal companionship: a crucial support for people during the COVID-19 pandemic. Society Register. (2020) 4:109–20. 10.14746/sr.2020.4.2.08

[37] Chadwin R. Evacuation of pets during disasters: a public health intervention to increase resilience. Am J Public Health. (2017) 107:1413–7. 10.2105/AJPH.2017.303877 28727532 PMC5551593

[38] Surkes S. The Times of Israel. More Pets Abandoned, More Adopted. Since Coronavirus Outbreak (2020). Available online at: https://www.timesofisrael.com/more-pets-abandoned-and-more-adopted-since-coronavirus-outbreak/ (accessed December 28, 2020).

[39] Flannigan G Dodman NG. Risk factors and behaviors associated with separation anxiety in dogs. J Am Vet Med Assoc. (2001) 219:360–466. 10.2460/javma.2001.219.460 11518171

[40] Irian M. COVID-19, your pet and other animals: are you at risk? Medic Rev. (2020) 22:81–2. 10.37757/MR2020.V22 33295324

[41] Sharun K Tiwari R Patel SK Karithik K Iqbal Yatoo M Malik YS. Coronavirus disease. (2020). (COVID-19) in domestic animals and wildlife: advances and prospects in the development of animal models for vaccine and therapeutic research. Hum Vaccin Immunother. (2019) 11:1–12. 10.1080/21645515.2020.1807802 PMC8641595 32915100

